# Use of direct oral anticoagulants in patients with atrial fibrillation in Scotland: Applying a coherent framework to drug utilisation studies

**DOI:** 10.1002/pds.4272

**Published:** 2017-07-28

**Authors:** Tanja Mueller, Samantha Alvarez‐Madrazo, Chris Robertson, Marion Bennie

**Affiliations:** ^1^ Strathclyde Institute of Pharmacy and Biomedical Sciences University of Strathclyde Glasgow UK; ^2^ Department of Mathematics and Statistics University of Strathclyde Glasgow UK; ^3^ Public Health and Intelligence Strategic Business Unit NHS National Services Scotland Edinburgh UK

**Keywords:** adherence, atrial fibrillation, discontinuation, DOAC, persistence, pharmacoepidemiology

## Abstract

**Purpose:**

To report the use of direct oral anticoagulants (DOACs) for stroke prevention in patients with atrial fibrillation in Scotland and advocate the standardisation of drug utilisation research methods.

**Methods:**

Retrospective cohort study using linked administrative data. Patients included those with a diagnosis of atrial fibrillation (confirmed in hospital) who received a first prescription for a DOAC (dabigatran, rivaroxaban, or apixaban) from September 2011 to June 2014. Drug utilisation measures included discontinuation, persistence, and adherence.

**Results:**

A total of 5398 patients (mean CHA_2_DS_2_‐VASc score 2.98 [SD 1.71], 89.7% with ≥5 concomitant medicines) were treated with DOACs for a median of 228 days (interquartile range 105‐425). Of 35.6% who discontinued DOAC treatment, 11.0% switched to warfarin, and 48.3% reinitiated DOACs. Persistence after 12 and 18 months was 75.9% and 69.8%, respectively. Differences between individual DOACs were observed: Discontinuation rates ranged from 20.4% (apixaban) to 60.6% (dabigatran) and 12 months persistence from 60.1% (dabigatran) to 85.5% (apixaban). Adherence to treatment with all DOACs was good: Overall DOAC median medication refill adherence was 102.9% (interquartile range 88.9%‐115.5%), and 82.3% of patients had a medication refill adherence > 80%.

**Conclusions:**

In Scotland, adherence to DOAC treatment was good, and switching from DOAC to warfarin was low. However, discontinuation and persistence rates were variable—although treatment interruptions were often temporary.

To decrease the inconsistencies in drug utilisation methods and facilitate meaningful study comparison, the use of a coherent framework—using a combination of discontinuation, persistence, and adherence—and the standardisation of measurements is advocated.

## 1 INTRODUCTION

Drug utilisation research (DUR) is frequently conducted to analyse the use of drugs, and a core aspect is adherence to drug treatment, defined as the extent to which patients take drugs according to prescribing instructions.^1^ Nonadherence to drugs is widespread^1^, ^2^, ^3^ and has been linked to increases in morbidity, premature death, and health care expenditure;^1^, ^4^, ^5^ especially nonadherence to drugs with complex pharmacological profiles such as warfarin and other vitamin K antagonists (VKAs) is known to negatively affect treatment outcomes.^6^, ^7^, ^8^, ^9^

Warfarin is used for multiple cardiovascular conditions, and high discontinuation rates of warfarin treatment have been reported in clinical trials and observational studies;^10^, ^11^, ^12^ poor adherence has been ascribed to a variety of issues, from the occurrence of bleeding events to the inconvenience of treatment.^6^, ^13^, ^14^, ^15^ As warfarin is widely used long‐term in patients with atrial fibrillation (AF)—a common condition causing irregular heartbeat and, as such, a major independent risk factor for stroke^16^, ^17^—efforts to replace warfarin have resulted in 4 new direct oral anticoagulants (DOACs) being introduced since 2008: dabigatran, a direct thrombin inhibitor, and rivaroxaban, apixaban, and edoxaban, direct factor Xa inhibitors.

Treatment with DOACs follows easier dosing schemes and theoretically requires no monitoring, primarily because their therapeutic windows are wider and interactions with other drugs/foods are shown to be less than with warfarin.^18^, ^19^ Treatment with DOACs is deemed as effective and safe as with warfarin;^20^, ^21^, ^22^, ^23^ the effect of nonadherence on bleeding risk and stroke incidence among AF patients has been addressed but as yet not intensely studied.^24^, ^25^ Recently, concerns have been raised about the potential impact of no monitoring and the presence of multimorbidity and polypharmacy on DOAC adherence.^24^, ^26^, ^27^, ^28^, ^29^ Knowledge about patients' adherence to DOACs in real life is however still limited, and studies conducted thus far differ in scale, methodology, and focus.^25^, ^30^, ^31^, ^32^, ^33^, ^34^, ^35^, ^36^, ^37^, ^38^, ^39^, ^40^, ^41^

Results emerging from DUR studies are frequently used to compare uptake and use of drugs over time and between regions. Additionally, information on treatment adherence can be used to improve the accuracy of drug exposure estimates when investigating treatment outcomes in clinical practice—as compared to clinical trials results.^42^ Nevertheless, although a comprehensive framework of drug adherence and a common terminology for measuring adherence have been proposed by the European Society for Patient Adherence, Compliance and Persistence,^43^ DUR studies still make use of a variety of definitions and measurements, with methods of calculation also differing.^44^, ^45^, ^46^ The objective of this study is therefore 2‐fold: first, to report on the use of DOACs for stroke prevention in patients with AF identified in Scottish secondary care; and second, to advocate the standardisation of DUR by applying an evolving methods approach, based on a sound theoretical framework and well‐documented measurements of adherence.

**KEY POINTS**

- Using Scottish national administrative data, we analysed the utilisation of the direct oral anticoagulants (DOACs) dabigatran, rivaroxaban, and apixaban in 5398 patients with atrial fibrillation between September 2011 and June 2014.
- Adherence to DOAC treatment was good, and switching from DOAC to warfarin was low. However, discontinuation and persistence rates were variable.
- Standardisation of drug utilisation studies using the ESPACOM framework, combining measurements of discontinuation, persistence, and adherence, is strongly advocated.

## 2 METHODS

In Scotland (population approximating 5.3 million^47^), all residents are covered by the publicly funded National Health Service (NHS), with most clinical services including prescriptions provided free of charge at the point of care.^48^

Prescription details have been derived from the Prescribing Information System (PIS)—an NHS Scotland national database created primarily for reimbursement purposes.^49^ The PIS holds data on the prescribed and dispensed items, patients, and prescribers, including a range of drug‐specific information based on the British National Formulary.^49^, ^50^ Clinical data have been extracted from the Scottish Morbidity Records/Hospital inpatients dataset (SMR01), comprising diagnostic codes according to the International Classification of Diseases, 10th edition, and Office of Population Censuses and Surveys procedural codes, 4th revision.^51^, ^52^, ^53^ PIS and SMR01 have been linked using the Community Health Index, a unique patient identifier issued to all residents registered with a general practitioner in Scotland.^54^

The study population comprises patients with a diagnosis of AF, confirmed in secondary care between January 1997 and June 2014, who received a DOAC between the date of a drug's approval for the indication of stroke prevention in patients with AF in Scotland (dabigatran: September 2011; rivaroxaban: January 2012; and apixaban: January 2013) and June 2014. For details about inclusion and exclusion criteria, see Appendix 1. A patient's index date for study inclusion was the date of first recorded prescription for any DOAC; their individual end date was either date of death or removal from a Scottish general practitioner register for other reasons, or the study end date (June 30, 2014), whichever occurred first. To assess baseline characteristics of patients, all available hospital records 5 years prior to the index date have been used. A time period of 6 months before the index date has been applied to define concomitant medication. Dispensed quantities and prescription dates have been used throughout the study.

Drug utilisation can be divided into 3 distinct parts: initiation, implementation, and discontinuation.^43^, ^55^ While initiation and discontinuation indicate the start and end of a therapy, the process of implementation illustrates whether medication was taken as prescribed. To give a valid representation of patients' drug taking behaviour and enable analysis of drug exposure, duration and intensity of treatment should be taken into account. Hence, this study includes measures of both discontinuation/persistence and adherence. Discontinuation has been calculated using the refill‐gap method with censoring of patients after the first discontinuation event,^56^ defined as a gap of more than 28 days without supply following the assumed end of a prescription, based on summary statistics of the data and comparable to previous studies.^30^, ^35^ In addition, a second discontinuation rate—allowing for reinitiation of treatment during the study period—has been introduced (cessation rate). In line with the literature, persistence at prespecified points in time (6, 12, and 18 months after treatment initiation) has been assessed using the anniversary method to account for intermediary treatment interruptions.^56^ As no “gold standard” to quantify adherence has been agreed upon, triangulation of measurements selected based on a literature review has been applied. See Table 1 for details. Drug utilisation measures are rations and are summarised using medians and interquartile ranges (IQRs).

**Table 1:** Utilisation measurements as used in this study, definitions, and calculation methods

Measurement		Definition	Calculation
Discontinuation rate^56^	Discontinuation rate (refill‐gap method)	Patients discontinuing treatment (ie, supply gap exceeding 28 days)	(Patients discontinuing treatment / patients initiating treatment) * 100
	Cessation rate (allowing for treatment interruptions)	Patients ceasing treatment (ie, no further prescription for any DOAC during the study period)	(Patients ceasing treatment / patients initiating treatment) * 100
Persistence rate^56^	Persistence after 6, 12, and 18 months (anniversary method)	Patients still on treatment 6, 12, and 18 months after initiation	(Patients with drug supply covering the anniversary date / patients with sufficient follow‐up time) * 100
Adherence^45^	Medication refill adherence (MRA)	Exposure to medication covering the time period of treatment	(Total days' supply / total days in study) * 100
	Compliance rate (CR)	Exposure to medication covering the time period of treatment	(Total days' supply—last refill) / days first up to, but not including last refill) * 100
	Continuous, single‐interval measure of medication availability (CSA)	Exposure to medication covering the time period between individual dispensations	(Days' supply per dispensing / days in dispensing interval) * 100

Kaplan‐Meier survival analysis has been used to calculate median time to discontinuation; follow‐up has been censored after 1 year for apixaban and after 2 years for dabigatran and rivaroxaban. Events have been coded as 1 = treatment discontinuation and 0 = still on treatment at end of follow‐up. All data analyses have been conducted using the R software, version 3.3.1.^57^

Because of the nature of the study, ethical approval was not required; however, use of the data has been approved by the appropriate Privacy Advisory Committee. The data were hosted and managed by the NHS Scotland National Safe Haven.

## 3 RESULTS

A total of 5398 patients were included in the study, 48.1% of whom used a VKA in the 6‐month period preceding DOAC treatment. Overall median follow‐up time was 228 days (IQR 105‐425), ranging from 124.5 days (IQR 54‐226.2) for apixaban to 467 days (IQR 237.8‐719.2) for dabigatran. The median number of DOAC prescriptions issued to patients was 5 (IQR 2‐9); 15.6% of patients received only one DOAC prescription.

Multimorbidity and polypharmacy were widespread. Most patients (89.7%) were treated with 5 or more different drugs concomitantly; most prevalent medications were beta‐blockers (66.5% of patients), statins (57.1%), drugs influencing the renin‐angiotensin system (55.4%), diuretics (51.9%), and analgesics other than non‐steroidal anti‐inflammatory drugs (50.3%). Prior to treatment initiation, drugs that should either be avoided or are contraindicated in combination with DOACs were prescribed to 5.0% and 2.2% of patients, respectively. Table 2 gives an overview of patients' baseline characteristics by first DOAC prescribed.

**Table 2:** Patients' baseline characteristics, overall and by first drug prescribed

	DOAC (n = 5398)	Dabigatran (n = 1016)	Rivaroxaban (n = 3292)	Apixaban (n = 1090)
Calendar year of first prescription (%)				
2011	51 (0.9)	51 (5.0)	0	0
2012	911 (16.9)	411 (40.5)	500 (15.2)	0
2013	2426 (44.9)	405 (39.9)	1624 (49.3)	397 (36.4)
2014	2010 (37.2)	149 (14.7)	1168 (35.5)	693 (63.6)
Female (%)	2472 (45.8)	400 (39.4)	1548 (47.0)	524 (48.1)
Mean age first prescription (SD)	74.4 (11.3)	71.6 (11.8)	75.3 (10.9)	74.3 (11.5)
Patient age category at time of first prescription (%)				
<50	169 (3.1)	44 (4.3)	80 (2.4)	45 (4.1)
50‐64	763 (14.1)	212 (20.9)	407 (12.4)	144 (13.2)
65‐74	1453 (26.9)	306 (30.1)	862 (26.2)	285 (26.1)
75‐84	2007 (37.2)	310 (30.5)	1279 (38.9)	418 (38.3)
85+	1006 (18.6)	144 (14.2)	664 (20.2)	198 (18.2)
Co‐morbidities as included in CHA_2_DS_2_‐VASc score (%)^a^				
Congestive heart failure	1007 (18.7)	163 (16.0)	608 (18.5)	236 (21.7)
Hypertension	2067 (38.3)	379 (37.3)	1268 (38.5)	420 (38.5)
Diabetes mellitus	840 (15.6)	151 (14.9)	533 (16.2)	156 (14.3)
Prior stroke/TIA	839 (15.5)	144 (14.2)	520 (15.8)	175 (16.1)
Vascular disease	547 (10.1)	96 (9.4)	347 (10.5)	104 (9.5)
Mean CHA_2_DS_2_‐VASc score (SD)	2.98 (1.71)	2.65 (1.74)	3.07 (1.70)	3.03 (1.65)
Prior VKA use yes (%)	2595 (48.1)	479 (47.1)	1644 (49.9)	472 (43.3)
Concomitant antiplatelet use (%)^b^	599 (11.1)	91 (9.0)	364 (11.1)	144 (13.2)
Concomitant aspirin use (%)^c^	1846 (34.2)	411 (40.5)	1039 (31.6)	396 (36.3)
Concomitant NSAID use (%)^b^	327 (6.1)	80 (7.9)	176 (5.3)	71 (6.5)
Concomitant use of contraindicated drugs (%)^d^	118 (2.2)	9 (0.9)	78 (2.4)	31 (2.8)
Concomitant use of drugs that should be avoided (%)^e^	268 (5.0)	53 (5.2)	147 (4.5)	68 (6.2)
Mean number different drugs prior to DOAC initiation (SD)	10.8 (5.5)	9.8 (5.2)	11.0 (5.5)	11.0 (5.5)

Abbreviations: DOAC, direct oral anticoagulant; NSAID, non‐steroidal anti‐inflammatory drug; SD, standard deviation; TIA, transient ischaemic attack; VKA, vitamin K antagonist.

a Based on hospital records only—ICD‐10 codes included in the CHA_2_DS_2_‐VASc score can be found in Appendix 2.

b Excluding aspirin.

c Includes only prescribed aspirin—potential underestimation of use as aspirin can be acquired over‐the‐counter in Scotland.

d Dabigatran: itraconazole, ketoconazole; rivaroxaban and apixaban: carbamazepine, itraconazole, ketoconazole, phenytoin, rifampicin.^50^

e Dabigatran: carbamazepine, clarithromycin, phenytoin; rivaroxaban and apixaban: clarithromycin.^50^

**Figure 1:**
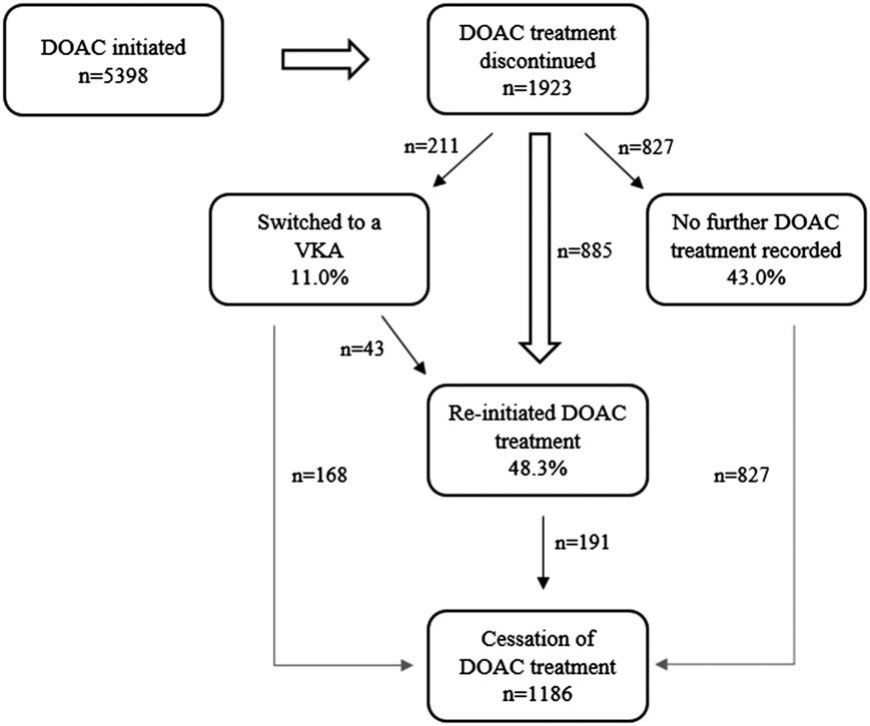
Patients' treatment options after DOAC discontinuation. DOAC, direct oral anticoagulant; VKA, vitamin K antagonist

When looking at DOACs in general—ie, disregarding switches between individual drugs—1923 patients (35.6%) discontinued treatment during the study period, and the median time to discontinuation was 393 days (95% CI, 374‐428 days); however, 48.3% of patients discontinuing reinitiated treatment with any DOAC at least temporarily (see Figure 1 for details). By study conclusion, 1186 patients had stopped receiving DOAC prescriptions, resulting in a cessation rate of 22.0%; this figure includes patients ceasing all oral anticoagulant treatment as well as those switching lastingly to a VKA.

**Figure 2:**
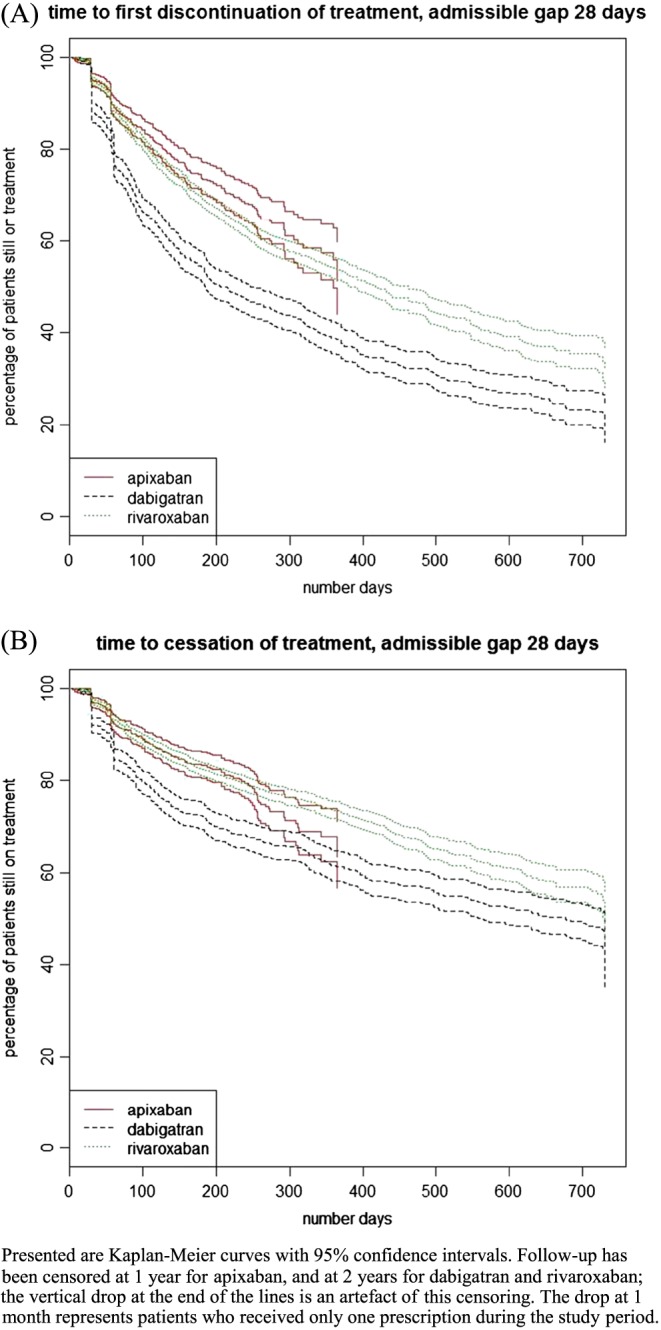
Kaplan‐Meier survival curves by drug [Colour figure can be viewed at wileyonlinelibrary.com]

For individual DOACs, a total of 1995 patients (37.0%) discontinued treatment with the first drug prescribed, and discontinuation rates differed substantially between drugs: 20.4% apixaban, 35.1% rivaroxaban, and 60.6% dabigatran. Median time to discontinuation was considerably shorter for dabigatran (206 days; 95% CI, 185‐247 days) than rivaroxaban (414 days; 95% CI, 382‐462). While 35.1% of apixaban patients who discontinued restarted treatment, proportions for dabigatran and rivaroxaban were 44.2% and 46.1%, respectively; accounting for reinitiations, the share of patients who eventually ceased treatment with their index drug ranged from 14.4% for apixaban to 42.4% for dabigatran. Survival curves of patients discontinuing treatment, regardless of permanency, in contrast to patients ceasing treatment, are shown in Figure 2.

Crude persistence with DOAC treatment regardless of switches between individual drugs was 82.1%, 75.9%, and 69.8% at 6, 12 and 18 months, respectively. Persistence for individual drugs at 6, 12, and 18 months was 68.9%, 60.1%, and 54.7% for dabigatran and 83.5%, 79.0%, and 74.9% for rivaroxaban. Persistence with apixaban was 86.8% at 6 months and 85.5% at 12 months; insufficient data were available at 18 months.

Extending the length of admissible gap without drug supply from 28 to 56 days considerably decreased the DOAC discontinuation rate to 20.0%, but had less of an impact on persistence rates; persistence increased at 6 months to 85.8% and at 12 months to 77.9%. Relative differences between individual DOACs were not affected. Additional results of the sensitivity analysis can be found in Appendix 3.

A total of 4555 patients (84.4%) received at least 2 DOAC prescriptions and have been included in calculations of adherence to treatment. All adherence measurements gave comparable results and indicate high adherence to DOAC treatment, albeit with differences between individual drugs. Adherence remained stable over time when looking at 6 months intervals rather than assessing patients' entire treatment periods; see Table 3 for details.

**Table 3:** Adherence to treatment, overall and by first drug prescribed

	DOAC (n = 4555)	Dabigatran (n = 864)	Rivaroxaban (n = 2821)	Apixaban (n = 822)
MRA > 80% (%)^a^	82.3	64.5	83.3	88.0
Median MRA (IQR)^a^	102.9 (88.9‐115.5)	95.1 (56.7‐107.1)	103.1 (90.3‐115.0)	107.2 (93.5‐124.7)
Median MRA over time (IQR)^b^				
0‐6 months	108.9 (93.3‐124.4)	100.0 (83.3‐116.7)	108.9 (93.3‐124.4)	111.7 (93.3‐124.4)
7‐12 months	112.0 (100.6‐125.4)	110.6 (98.6‐124.2)	111.5 (101.2‐125.3)	111.7 (98.8‐123.5)
13‐18 months	112.0 (101.2‐126.4)	112.0 (98.9‐125.9)	112.0 (103.1‐126.3)	n/a
CR > 80% (%)^a^	90.6	84.6	92.3	91.4
Median CR (IQR)^a^	103.5 (95.1‐115.3)	100.6 (90.7‐110.0)	103.7 (96.2‐115.9)	104.7 (96.0‐121.4)
Median CR over time (IQR)^b^				
0‐6 months	105.3 (96.0‐117.2)	103.5 (92.0‐115.4)	105.7 (96.6‐117.5)	104.2 (96.0‐115.5)
7‐12 months	100.0 (94.1‐108.4)	100.7 (92.3‐109.4)	100.0 (94.1‐107.7)	100.0 (96.1‐107.1)
13‐18 months	100.8 (94.0‐108.5)	101.4 (90.9‐110.1)	100.6 (95.2‐108.4)	n/a
Median CSA (IQR)^a^	100.0 (90.3‐122.4)	100.0 (87.0‐120.0)	100.0 (90.3‐124.4)	103.4 (88.9‐130.4)
Median CSA over time (IQR)^b^				
0‐6 months	103.7 (90.3‐133.3)	103.4 (88.2‐130.4)	103.7 (90.3‐133.3)	103.7 (90.3‐133.3)
7‐12 months	100.0 (90.3‐116.7)	101.7 (88.2‐120.0)	100.0 (90.3‐116.7)	100.0 (88.7‐112.0)
13‐18 months	100.0 (90.3‐116.7)	100.0 (88.2‐117.6)	100.0 (90.3‐116.7)	n/a

Abbreviations: CR, compliance rate; CSA, continuous, single‐interval measure of medication availability; DOAC, direct oral anticoagulant; IQR, interquartile range; MRA, medication refill adherence.

To calculate adherence, only patients who received at least 2 prescriptions have been included. Discrepancies between the total number of patients receiving 2 prescriptions for any DOAC (n = 4555) and the sum of patients receiving at least 2 prescriptions for dabigatran, rivaroxaban, or apixaban (n = 4507) are due to patients switching drugs after only one prescription for an initial drug.

a Includes all patients with at least 2 prescriptions during the study period.

b Includes only patients with sufficient follow‐up time to cover the respective prescription period.

## 4 DISCUSSION

This is the first study in Scotland using linked data from PIS and SMR01 to analyse discontinuation, persistence, and adherence to DOAC treatment and one of a small number of studies analysing routinely collected data at a national level.^25^, ^33^ Additionally, it is one of the first studies to apply the European Society for Patient Adherence, Compliance and Persistence framework for drug utilisation studies^43^ in combination with proposed standardised measurements for drug adherence;^45^ it encompasses all dimensions of drug utilisation rather than focusing on a single aspect and therefore gives a much more comprehensive picture of how DOACs are used in clinical practice than previous studies.

Our findings are, at large, comparable to other research: AF patients being treated with DOACs are in general elderly, have high levels of co‐morbidities, and receive a large number of additional drugs;^31^, ^32^, ^34^, ^38^, ^58^ discontinuation of treatment varies considerably between individual DOACs;^30^, ^32^, ^38^, ^40^ and persistence declines over time.^32^, ^35^ Nevertheless, two important differences need to be highlighted: First, adherence to treatment was considerably higher in our study than has been reported in other studies conducted on a national level;^25^, ^33^ and second, switches from DOACs to VKAs were much less common than in previous observational studies.^31^, ^36^, ^38^

The crude discontinuation rate and 12 months persistence to DOAC treatment in Scotland were 35.6% and 75.9%, respectively, and similar rates have been reported before;^35^, ^41^ however, most studies reporting on discontinuation and/or persistence either did not explicitly describe how rates have been calculated or did not clearly distinguish between the different concepts of discontinuation and persistence. In our study, 48.3% of patients discontinuing treatment subsequently received at least one additional prescription for any DOAC, ie, eventually resumed treatment. When allowing for treatment interruptions by using only the number of patients where no subsequent DOAC prescriptions were recorded—a figure considerably influenced by length of follow‐up, as more patients might reinitiate treatment over time—the crude DOAC discontinuation rate was 22.0%, highlighting that the method of calculation where patients are censored after the occurrence of a first discontinuation event likely leads to an overestimation of discontinuation. This lower discontinuation rate is also more in agreement with the persistence rates found in this study, calculated using the anniversary method that is insensitive to periods of treatment interruptions.^56^

The percentage of patients switching from a DOAC to VKA treatment was low in this study (11.0%) compared to patients in Denmark (51.2%) and Japan (54%), but higher than in England (6.0%), and similar to the 1‐year follow‐up EORP‐AF pilot registry (11.8%);^35^, ^36^, ^38^, ^59^ the reasons for these diverse findings are unclear, but might be rooted either in differences in clinical guidelines and physicians' preferences, or in timing of studies and availability of DOACs.

Although many studies report on DOACs as a group rather than separately by individual drugs, our results confirm previous findings indicating sizable differences between individual drugs: Dabigatran frequently exhibits discontinuation rates higher than rivaroxaban and apixaban.^32^, ^38^ Consequently, 12 months persistence has reportedly been lowest for dabigatran, ranging from 44.7% to 74.4% compared to 60.1% to 77.4% for rivaroxaban and up to 85.9% for apixaban^32^, ^34^—results that match our own findings of 60.1% (dabigatran), 79.0% (rivaroxaban), and 85.5% (apixaban).

Discontinuation of DOAC treatment has often been attributed to changes in underlying disease severity, including restoration of sinus rhythm, worsening kidney function, and side effects including bleeding events or, particularly in case of dabigatran, gastrointestinal disturbances.^31^, ^38^, ^41^ Because of the data available for this study, specific reasons for treatment discontinuations among the study population remain unknown.

Adherence to medication was high for all drugs and did not considerably decline over time. Treatment gaps were rare, patients generally having enough medication to cover the treatment period; median medication refill adherence, and compliance rate during the first 6 months of treatment, indicated oversupply rather than undersupply of drugs, although these findings might be due to the timing of prescriptions. High median adherence to DOAC treatment has been shown before: between 94% and 99.7%^37^, ^39^ for dabigatran and up to 100% for rivaroxaban and apixaban.^30^ Results differ however, which might in part be due to distinct methodology, as calculation methods for adherence and analysable time periods varied between studies. Moreover, results are frequently reported in dichotomised form, with a threshold of 80% of the calculated measurement used to identify adherent patients; findings using this approach range from 38.5% to 92.0% for dabigatran,^25^, ^33^, ^34^, ^37^, ^39^ 50.5% to 96% for rivaroxaban,^25^, ^30^, ^34^ and 61.9% to 95% for apixaban^25^, ^30^, ^34^—placing our findings of the proportion of patients with a compliance rate > 80% of 84.6%, 92.3%, and 91.4% for dabigatran, rivaroxaban, and apixaban, respectively, at the upper end of each scale. Comparing our findings obtained with 3 different adherence measurements, applied to the study data, illustrated how differences in calculation methods can impact the results; these observations raise concerns regarding comparability and generalisability of findings, especially when methods are not clearly described.

This study has a number of limitations. First, by identifying eligible patients to be included in the study in secondary care, patients diagnosed and treated exclusively in primary care were not captured. A recent study, conducted in England, identified that 42.1% of AF patients had an initial diagnosis in primary care;^60^ however, as many of these patients are relatively elderly, a proportion might subsequently be admitted to hospital, and therefore, the percentage of patients potentially not included in our study is likely to be lower than 40%. We do not anticipate this having a large impact on our findings regarding adherence, persistence, and discontinuation. Second, the data used for analysis have not been collected for the specific purpose of this study but were gathered routinely in daily care. Therefore, not all desirable information was present; in particular, no indication for why drugs have been prescribed has been available. This might have resulted in the inclusion of patients who had AF but were treated with DOACs for other reasons—specifically, treatment with any DOAC for up to 6 months due to deep vein thrombosis—potentially leading to imprecision in results due to diverging anticipated treatment lengths and dosing schedules. In Figure 2, there is a small drop at 180 days of only about 3%, so the impact of this on persistence is likely to be small. Dose instructions as recorded by the prescriber have been used supplementary to drug supply based on standard dosing guidelines to limit the impact of variations in dosing schedules on adherence. Finally, as prescription records do not cover secondary care, in‐patient periods are not included; this might have impacted adherence and persistence, as hospital days could have appeared to be treatment interruptions. Sensitivity analysis of the lengths of admissible gaps and the additional measurement of treatment cessation have been used to account for the potential effect of in‐patient episodes on discontinuation and persistence.

This study has nevertheless several strengths: Access to health care is universal, and electronic health records in Scotland cover the entire population. Because of the presence of a unique patient identifier, records can easily and reliably be linked; a large variety of variables is therefore available, including those essential for calculating adherence to medication. Furthermore, PIS and SMR01 have previously been used for research, and validity and accuracy of the data has been established.^49^, ^52^

## 5 CONCLUSION

In Scotland, adherence to DOAC treatment was high, and switching from DOAC to warfarin was low. However, discontinuation and persistence rates were variable—although treatment interruptions were often temporary. The effects of nonadherence, including treatment interruptions, on the safety and effectiveness of DOACs need to be investigated further; more research is needed to analyse whether treatment with DOACs does indeed result in better disease outcomes as compared to warfarin.

To decrease the inconsistencies in drug utilisation methodology impacting the comparability of results across studies, the use of a coherent framework—using a combination of discontinuation, persistence, and adherence—and the standardisation of measurements is strongly advocated.

## ETHICS STATEMENT

The authors state that no ethical approval was needed.

## CONFLICT OF INTEREST

The authors declare no conflict of interest

## Supporting information

Appendix 1: Cohort identification and selection of study population
Appendix 2: ICD‐10 codes as used for calculation of CHA_2_DS_2_‐VASc scores at baseline
Appendix 3: Sensitivity analysis discontinuation and persistence

## References

[1] WHO . Adherence to Long‐term Therapies: Evidence for Action. Geneva: World Health Organization; 2003.

[2] Kolandaivelu K , Leiden BB , O'Gara PT , Bhatt DL . Non‐adherence to cardiovascular medications. Eur Heart J. 2014;35:3267‐3276. https://doi.org/10.1093/eurheartj/ehu364 25265973 10.1093/eurheartj/ehu364

[3] Schulz M , Krueger K , Schuessel K , et al. Medication adherence and persistence according to different antihypertensive drug classes: a retrospective cohort study of 255,500 patients. Int Journal of Cardiol. 2016;220:668‐676. https://doi.org/10.1016/j.ijcard.2016.06.263 10.1016/j.ijcard.2016.06.263 27393848

[4] Bansilal S , Castellano JM , Garrido E , et al. Assessing the impact of medication adherence on long‐term cardiovascular outcomes. J Am Coll Cardiol. 2016;68:789‐801. https://doi.org/10.1016/j.jacc.2016.06.005 27539170 10.1016/j.jacc.2016.06.005

[5] Ho PM , Bryson CL , Rumsfeld JS . Medication adherence: its importance in cardiovascular outcomes. Circulation. 2009;119:3028‐3035. https://doi.org/10.1161/CIRCULATIONAHA.108.768986 19528344 10.1161/CIRCULATIONAHA.108.768986

[6] Cotté FE , Benhaddi H , Duprat‐Lomon I , et al. Vitamin K antagonist treatment in patients with atrial fibrillation and time in therapeutic range in four European countries. Clin Ther. 2014;36:1160‐1168. https://doi.org/10.1016/j.clinthera.2014.07.016 25151574 10.1016/j.clinthera.2014.07.016

[7] Morgan CL , McEwan P , Tukiendorf A , Robinson PA , Clemens A , Plumb JM . Warfarin treatment in patients with atrial fibrillation: observing outcomes associated with varying levels of INR control. Thromb Res. 2009;124:37‐41. https://doi.org/10.1016.j.thromres.2008.09.016 19062079 10.1016/j.thromres.2008.09.016

[8] Schein JR , White CM , Nelson WW , Kluger J , Mearns ES , Coleman CI . Vitamin K antagonist use: evidence of the difficulty of achieving and maintaining target INR range and subsequent consequences. Thromb J. 2016;14:14 https://doi.org/10.1186/s12959‐016‐0088‐y 27303213 10.1186/s12959-016-0088-y PMC4906845

[9] Sherwood MW , Douketis JD , Patel MR , et al. Outcomes of temporary interruption of rivaroxaban compared with warfarin in patients with nonvalvular atrial fibrillation. Circulation. 2014;129:1850‐1859. https://doi.org/10.1161/CIRCULATIONAHA.113.005754 24552831 10.1161/CIRCULATIONAHA.113.005754 PMC4206548

[10] Ewen S , Rettig‐Ewen V , Mahfoud F , Boehm M , Laufs U . Drug adherence in patients taking oral anticoagulation therapy. Clin Res Cardiol. 2014;103:173‐182. https://doi.org/10.1007/s00392‐013‐0616‐8 23999974 10.1007/s00392-013-0616-8

[11] Fang MC , Go AS , Pomernacki NK , et al. Warfarin discontinuation after starting warfarin for atrial fibrillation. Circ Cardiovasc Qual Outcomes. 2010;3:624‐631. https://doi.org/10.1161/CIRCOUTCOMES.110.937680 20959565 10.1161/CIRCOUTCOMES.110.937680 PMC3063305

[12] O'Brien EC , Simon DN , Allen LA , et al. Reasons for warfarin discontinuation in the outcomes registry for better informed treatment of atrial fibrillation (ORBIT‐AF*)* . Am Heart J. 2014;168:487‐494. https://doi.org/10.1016/j.ahj.2014.07.002 25262258 10.1016/j.ahj.2014.07.002

[13] Lip GYH , Shantsila E . Handbook of Oral Anticoagulation. Heidelberg: Springer Healthcare; 2013.

[14] Nutescu E , Chuatrisorn I , Hellenbart E . Drug and dietary interactions of warfarin and novel oral anticoagulants: an update. J Thromb Thrombolysis. 2011;31:326‐343. https://doi.org/10.1007/s11239‐011‐0561‐1 21359645 10.1007/s11239-011-0561-1

[15] Kaariainen M , Paukama M , Kyngas H . Adherence with health regimens of patients on warfarin therapy. J Clin Nurs. 2013;22:89‐96. https://doi.org/10.1111/j.1365‐2702.2012.04079.x 22784012 10.1111/j.1365-2702.2012.04079.x

[16] McCartney DE , Lomas O , Cahill TJ . Atrial fibrillation. InnovAiT. 2015;8:485‐492. https://doi.org/10.1177/1755738014541425

[17] Wolf PA , Abbott RD , Kannel WB . Atrial fibrillation as an independent risk factor for stroke: The Framingham Study. Stroke. 1991;22:983‐988. 1866765 10.1161/01.str.22.8.983

[18] Deitelzweig S . Practical considerations in the use of novel oral anticoagulants for stroke prevention in nonvalvular atrial fibrillation. Cardiovasc Ther. 2014;32:74‐81. https://doi.org/10.1111/1755‐5922.12048 24119252 10.1111/1755-5922.12048

[19] Scaglione F . New oral anticoagulants: comparative pharmacology with vitamin K antagonists. Clin Pharmacokinet. 2013;52:69‐82. https://doi.org/10.1007/s40262‐012‐0030‐9 23292752 10.1007/s40262-012-0030-9

[20] Giugliano RP , Ruff CT , Braunwald E , et al. Edoxaban versus warfarin in patients with atrial fibrillation. N Engl J Med. 2013;369:2093‐2104. https://doi.org/10.1056/NEJMoa1310907 24251359 10.1056/NEJMoa1310907

[21] Granger CB , Alexander JH , McMurray JJ , et al. Apixaban versus warfarin in patients with atrial fibrillation. N Engl J Med. 2011;365:981‐992. https://doi.org/10.1056/NEJMoa1107039 21870978 10.1056/NEJMoa1107039

[22] Connolly SJ , Ezekowitz MD , Yusuf S , et al. Dabigatran versus warfarin in patients with atrial fibrillation. N Engl J Med. 2009;361:1139‐1151. https://doi.org/10.1056/NEJMoa0905561 19717844 10.1056/NEJMoa0905561

[23] Patel MR , Mahaffey KW , Garg J , et al. Rivaroxaban versus warfarin in nonvalvular atrial fibrillation. New Engl J Med. 2011;365:883‐891. https://doi.org/10.1056/NEJMoa1009638 21830957 10.1056/NEJMoa1009638

[24] Sanfélix‐Gimeno G , Rodriguez‐Bernal CL , Hurtado I , Baixauli‐Perez C , Librero J , Peiro O . Adherence to oral anticoagulants in patients with atrial fibrillation—a population‐based retrospective cohort study linking health information systems in the Valencia region, Spain: a study protocol. BMJ Open. 2015;5 https://doi.org/10.1136/bmjopen‐2015‐007613 10.1136/bmjopen-2015-007613 PMC4611755 26482766

[25] Yao X , Abraham N , Alexander GC , et al. Effect of adherence to oral anticoagulants on risk of stroke and major bleeding among patients with atrial fibrillation. J Am Heart Assoc. 2016;5 https://doi.org/10.1161/JAHA.115.003074 10.1161/JAHA.115.003074 PMC4802483 26908412

[26] Di Minno A , Spadarella G , Tufano A , Prisco D , Di Minno G . Ensuring medication adherence with direct oral anticoagulant drugs: lessons from adherence with vitamin K antagonists (VKAs). Thromb Res. 2014;133:699‐704. https://doi.org/10.1016/j.thromres.2014.01.016 24525314 10.1016/j.thromres.2014.01.016

[27] Jaspers Focks J , Brouwer MA , Wojdyla DM , et al. Polypharmacy and effects of apixaban versus warfarin in patients with atrial fibrillation: post hoc analysis of the ARISTOTLE trial. BMJ. 2016;353 https://doi.org/10.1136/bmj.i2868 10.1136/bmj.i2868 PMC4908974 27306620

[28] Piccini JP , Hellkamp AS , Washam JB , et al. Polypharmacy and the efficacy and safety of rivaroxaban versus warfarin in the prevention of stroke in patients with nonvalvular atrial fibrillation. Circulation. 2016;133:352‐360. https://doi.org/10.1161/CIRCULATIONAHA.115.018544 26673560 10.1161/CIRCULATIONAHA.115.018544

[29] Rodriguez RA , Carrier M , Wells PS . Non‐adherence to new oral anticoagulants: a reason for concern during long‐term anticoagulation? J Thromb Haemost. 2013;11:390‐394. https://doi.org/10.1111/jth.12086 23206117 10.1111/jth.12086

[30] Al‐Khalili F , Lindström C , Benson L . Adherence to anticoagulant treatment with apixaban and rivaroxaban in a real‐world setting. Clin Trials Regul Sci Cardiol. 2016;18:1‐4. https://doi.org/10.1016/j.ctrsc.2016.03.003

[31] Beyer‐Westendorf J , Förster K , Ebertz F , et al. Drug persistence with rivaroxaban therapy in atrial fibrillation patients‐results from the Dresden non‐interventional oral anticoagulation registry. Europace. 2015;17:530‐538. https://doi.org/10.1093/europace/euu319 25694537 10.1093/europace/euu319 PMC4381834

[32] Coleman CI , Tangirala M , Evers T , Pizzi C . Treatment persistence and discontinuation with rivaroxaban, dabigatran, and warfarin for stroke prevention in patients with non‐valvular atrial fibrillation in the United States. PLoS One. 2016;11 https://doi.org/10.1371/journal.pone.0157769 10.1371/journal.pone.0157769 PMC4915663 27327275

[33] Gorst‐Rasmussen A , Skjøth F , Larsen TB , Rasmussen LH , Lip GYH , Lane DA . Dabigatran adherence in atrial fibrillation patients during the first year after diagnosis: a nationwide cohort study. J Thromb Haemost. 2015;13:495‐504. https://doi.org/10.1111/jth.12845 25594442 10.1111/jth.12845

[34] Forslund T , Wettermark B , Hjemdahl P . Comparison of treatment persistence with different oral anticoagulants in patients with atrial fibrillation. Eur J Clin Pharmacol. 2016;72:329‐338. https://doi.org/10.1007/s00228‐015‐1983‐z 26613954 10.1007/s00228-015-1983-z

[35] Martinez C , Katholing A , Wallenhorst C , Freedman SB . Therapy persistence in newly diagnosed non‐valvular atrial fibrillation treated with warfarin or NOAC. A cohort study. Thromb Haemost. 2016;115:31‐39. https://doi.org/10.1160/TH15‐04‐0350 26246112 10.1160/TH15-04-0350

[36] Pottegård A , Poulsen BK , Larsen MD , Hallas J . Dynamics of vitamin K antagonist and new oral anticoagulants use in atrial fibrillation: a Danish drug utilization study. J Thromb Haemost. 2014;12:1413‐1418. https://doi.org/10.1111/jth.12662 25039280 10.1111/jth.12662

[37] Schulman S , Shortt B , Robinson M , Eikelboom JW . Adherence to anticoagulant treatment with dabigatran in a real‐world setting. J Thromb Haemost. 2013;11:1295‐1299. https://doi.org/10.1111/jth.12241 23855420 10.1111/jth.12241

[38] Shiga T , Naganuma M , Nagao T , et al. Persistence of non‐vitamin K antagonist oral anticoagulant use in Japanese patients with atrial fibrillation: a single‐center observational study. J Arrhythm. 2015;31:339‐344. https://doi.org/10.1016/j.joa.2015.04.004 26702312 10.1016/j.joa.2015.04.004 PMC4672038

[39] Shore S , Carey EP , Turakhia MP , et al. Adherence to dabigatran therapy and longitudinal patient outcomes: insights from the Veterans Health Administration. Am Heart J. 2014;167:810‐817. https://doi.org/10.1016/j.ahj.2014.03.023 24890529 10.1016/j.ahj.2014.03.023 PMC5381802

[40] Simons LA , Ortiz M , Freeman SB , Waterhouse BJ , Colquhoun D , Thomas G . Improved persistence with non‐vitamin‐K oral anticoagulants compared with warfarin in patients with atrial fibrillation: recent Australian experience. Curr Med Res Opin. 2016;32:1857‐1861. https://doi.org/10.1080/03007995.2016.1218325 27463735 10.1080/03007995.2016.1218325

[41] Thorne K , Jayathissa S , Dee S , et al. Adherence and outcomes of patients prescribed dabigatran (Pradaxa) in routine clinical practice. Intern Med J. 2014;44:261‐265. https://doi.org/10.1111/imj.12370 24405800 10.1111/imj.12370

[42] Lee D , Bergmann U . Studies of drug utilization In: Strom BL, Kimmel SE, Hennessy S, eds. Pharmacoepidemiology. Wiley Blackwell: Chichester; 2012:379‐401.

[43] Vrijens B , De Geest S , Hughes DA , et al. A new taxonomy for describing and defining adherence to medications. Br J Clin Pharmacol. 2012;73:691‐705. https://doi.org/10.1111/j.1365‐2125.2012.04167.x 22486599 10.1111/j.1365-2125.2012.04167.x PMC3403197

[44] Caetano PA , Lam JMC , Morgan SG . Toward a standard definition and measurement of persistence with drug therapy: examples from research on statin and antihypertensive utilization. Clinl Ther. 2006;28:1411‐1424. https://doi.org/10.1016/j.clinthera.2006.09.021 10.1016/j.clinthera.2006.09.021 17062314

[45] Hess LM , Raebel MA , Connor DA , Malone DC . Measurement of adherence in pharmacy administrative databases: a proposal for standard definitions and preferred measures. Ann Pharmacother. 2006;40:1280‐1288. https://doi.org/10.1345/aph.1H018 16868217 10.1345/aph.1H018

[46] Lehmann A , Aslani P , Ahmed R , et al. Assessing medication adherence: options to consider. Int J Clin Pharmacol. 2014;36:55‐69. https://doi.org/10.1007/s11096‐013‐9865‐x 10.1007/s11096-013-9865-x 24166659

[47] NRS . Mid‐year population estimates. Edinburgh; 2016 http://www.nrscotland.gov.uk/statistics‐and‐data/statistics/statistics‐by‐theme/population/population‐estimates/mid‐year‐population‐estimates (accessed 14 December 2016).

[48] NHS Scotland . Your health, your rights. Edinburgh; 2016 http://www.nhsinform.org.uk/rights/usingnhs/access/rights/ (accessed 14 December 2016).

[49] Alvarez‐Madrazo S , McTaggart S , Nangle C , Nicholson E , Bennie M . Data resource profile: the Scottish National Prescribing Information System (PIS). Int J Epidemiol. 2016;45:714‐715f. https://doi.org/10.1093/ije/dyw060 27165758 10.1093/ije/dyw060 PMC5005947

[50] Joint Formulary Committee . British National Formulary BNF 71. London: BMJ Group Pharmaceutical Press; 2016.

[51] ISD Scotland . Coding and terminology systems. Edinburgh; 2010 http://www.isdscotland.org/Products‐and‐Services/Terminology‐Services/Coding‐and‐Terminology‐Systems/ (accessed 14 December 2016).

[52] ISD Scotland . National Data Catalogue: National Datasets. Edinburgh; 2016 http://www.ndc.scot.nhs.uk/National‐Datasets/ (accessed 14 December 2016).

[53] WHO . International Statistical Classification of Diseases and Related Health Problems 10th Revision. Geneva; 2016 http://apps.who.int/classifications/icd10/browse/2016/en (accessed 14 December 2016).

[54] ISD Scotland . ISD Scotland data dictionary. Edinburgh; 2016 http://www.ndc.scot.nhs.uk/Dictionary-A-Z/Definitions/index.asp?Search=C%26ID=128%26Title=CHI%20Number (accessed 14 December 2016).

[55] Vrijens B . An introduction to adherence research In: Elseviers M, Wettermark B, Almarsdottir AB, et al., eds. Drug Utilisation Research: Methods and Applications. Wiley Blackwell: Chichester; 2016:355‐360.

[56] Gregoire JP , Moisan J . Assessment of adherence to drug treatment in database research In: Elseviers M, Wettermark B, Almarsdottir AB, et al., eds. Drug Utilization Research: Methods and Applications. Wiley Blackwell: Chichester; 2016:369‐380.

[57] R Core Team . R: a language and environment for statistical computing. R Foundation for Statistical Computing: Vienna, 2016.

[58] Olesen JB , Sørensen R , Hansen ML , et al. Non‐vitamin K antagonist oral anticoagulation agents in anticoagulant naïve atrial fibrillation patients: Danish nationwide descriptive data 2011‐2013. Europace. 2015;17:187‐193. https://doi.org/10.1093/ije/dyw060 25236181 10.1093/europace/euu225

[59] Lip GYH , Laroche C , Ioachim PM , et al. Prognosis and treatment of atrial fibrillation patients by European cardiologists: one year follow‐up of the EURObservational Research Programme–Atrial Fibrillation General Registry Pilot Phase (EORP‐AF pilot registry). Eur Heart J. 2014;35:3365‐3376. https://doi.org/10.1093/eurheartj/ehu374 25176940 10.1093/eurheartj/ehu374

[60] Allan V , Banerjee A , Shah AD , et al. Net clinical benefit of warfarin in individuals with atrial fibrillation across stroke risk and across primary and secondary care. Heart. 2016;0:1‐9. https://doi.org/10.1136/heartjnl‐2016‐309910 10.1136/heartjnl-2016-309910 PMC5284481 27580623

