# Supplementary material for: Use of direct oral anticoagulants in patients with atrial fibrillation in Scotland: Applying a coherent framework to drug utilisation studies
Source: Pharmacoepidemiol Drug Saf. 2017 Jul 28;26(11):1378–86. doi: 10.1002/pds.4272 (PMC5697642; doi:10.1002/pds.4272)
Supplement: Supplementary file 1 — Appendix 1: Cohort identification and selection of study population Appendix 2: ICD‐10 codes as used for calculation of CHA2DS2‐VASc scores at baseline Appendix 3: Sensitivity analysis discontinuation and persistence [file PDS-26-1378-s001.docx]

**Appendix**


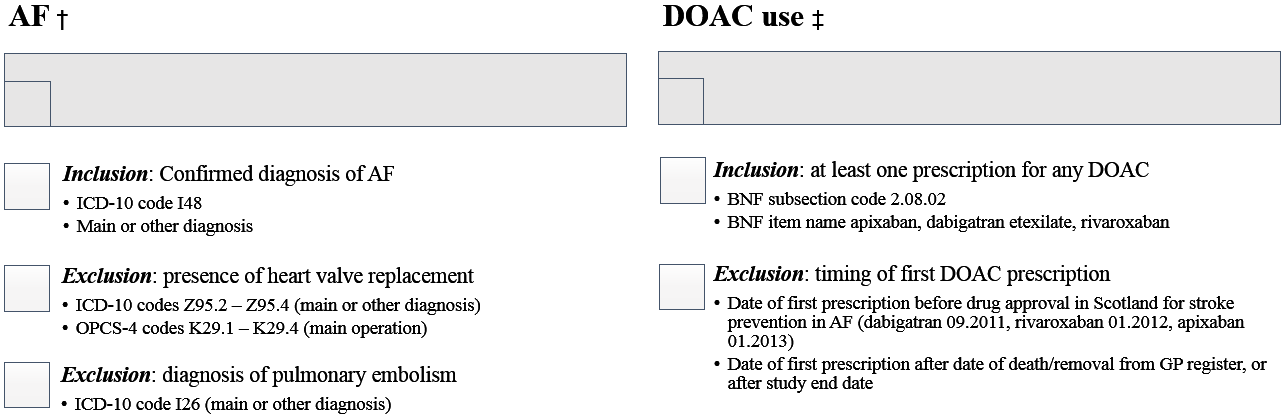


AF – atrial fibrillation; BNF – British National Formulary; GR – General Practitioner; DOAC – direct oral anticoagulant; ICD-10: International Classification of Diseases, 10^th^ edition; OPCS-4: Office of Population Censuses and Surveys procedural codes, 4^th^ revision

† Extracted from Scottish Morbidity Records/Hospital inpatients dataset (SMR01).

‡ Extracted from Prescribing Information System (PIS).

Appendix 1: Cohort identification and selection of study population


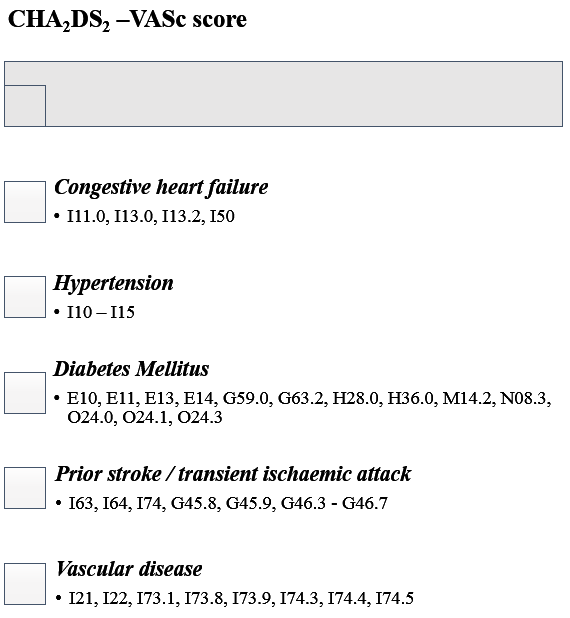


Appendix 2: ICD-10 codes as used for calculation of CHA_2_DS_2_-VASc scores at baseline

Appendix 3: Sensitivity analysis discontinuation and persistence

|  | DOAC | Dabigatran | Rivaroxaban | Apixaban |
| --- | --- | --- | --- | --- |
| Admissible gap: 28 days | | | | |
| *Discontinuation rate* | 35.6%  (n=5398) | 60.6%  (n=1016) | 35.1%  (n=3292) | 20.4%  (n=1090) |
| *Cessation rate* | 22.0%  (n=5398) | 42.4%  (n=1016) | 22.0%  (n=3292) | 14.4%  (n=1090) |
| *Persistence after 6 months* | 82.1%  (n=3214) | 68.9%  (n=830) | 83.5%  (n=2004) | 86.8%  (n=380) |
| *Persistence after 12 months* | 75.9%  (n=1771) | 60.1%  (n=642) | 79.0%  (n=1074) | 85.5%  (n=55) |
| *Persistence after 18 months* | 69.8%  (n=858) | 54.7%  (n=415) | 74.9%  (n=443) | n/a |
| Admissible gap: 56 days |  |  |  |  |
| *Discontinuation rate* | 20.0%  (n=5398) | 44.4%  (n=1016) | 19.2%  (n=3292) | 8.4%  (n=1090) |
| *Cessation rate* | 14.5%  (n=5398) | 35.5%  (n=1016) | 14.3%  (n=3292) | 6.9%  (n=1090) |
| *Persistence after 6 months* | 85.8%  (n=3214) | 74.6%  (n=830) | 86.7%  (n=2004) | 90.3%  (n=380) |
| *Persistence after 12 months* | 77.9%  (n=1771) | 63.1%  (n=642) | 80.3%  (n=1074) | 90.9%  (n=55) |
| *Persistence after 18 months* | 72.3%  (n=858) | 57.8%  (n=415) | 77.0%  (n=443) | n/a |
| Admissible gap: 84 days |  |  |  |  |
| *Discontinuation rate* | 15.1%  (n=5398) | 36.8%  (n=1016) | 14.6%  (n=3292) | 4.8%  (n=1090) |
| *Cessation rate* | 12.4%  (n=5398) | 31.9%  (n=1016) | 12.3%  (n=3292) | 4.7%  (n=1090) |
| *Persistence after 6 months* | 88.6%  (n=3214) | 79.0%  (n=830) | 89.4%  (n=2004) | 92.4%  (n=380) |
| *Persistence after 12 months* | 78.9%  (n=1771) | 64.0%  (n=642) | 81.5%  (n=1074) | 90.9%  (n=55) |
| *Persistence after 18 months* | 73.0%  (n=858) | 58.3%  (n=415) | 77.9%  (n=443) | n/a |
| n – number of patients included in each respective category: comprising all patients initiating treatment with a specific drug for calculation of discontinuation rates; and comprising patients with sufficient follow-up time for persistence rates after pre-specified periods of time | | | | |
